# Curative Effects of Oleanolic Acid on Formed Hypertrophic Scars in the Rabbit Ear Model

**DOI:** 10.1155/2012/837581

**Published:** 2012-12-27

**Authors:** Hong Zhang, Yan Zhang, Yi-Ping Jiang, Lan-Ke Zhang, Cheng Peng, Kun He, Khalid Rahman, Lu-Ping Qin

**Affiliations:** ^1^Department of Pharmacognosy, School of Pharmacy, Second Military Medical University, Shanghai 200433, China; ^2^Key Laboratory of Standardization of Chinese Herbal Medicines of Ministry of Education, Pharmacy College, Chengdu University of Traditional Chinese Medicine, Chengdu 610075, China; ^3^Department of Pharmacology, School of Pharmacy, Ningxia Medical University, Yinchuan 750004, China; ^4^Shanghai Anshan Experimental Middle School, Shanghai 200433, China; ^5^The School of Pharmacy & Biomolecular Sciences, Faculty of Science, Liverpool John Moores University, Liverpool L3 3AF, UK

## Abstract

Hypertrophic scarring is a common proliferative disorder of dermal fibroblasts characterized by collagen overproduction and excessive deposition of extracellular matrix (ECM). There is no consensus about the best therapeutics to produce complete and permanent improvement of scars with few side effects. To investigate the therapeutic effects of oleanolic acid (OA) on hypertrophic scars and explore the possible mechanism of action involved, a rabbit ear model with hypertrophic scars was established. OA (2.5%, 5%, and 10%) was given once daily to the scars for 28 consecutive days. As a result, OA significantly alleviated formed hypertrophic scars on rabbit ears. The levels of TGF-*β*_1_, MMP-1, TIMP-1, and collagens I and III were notably decreased, and the number of apoptosis cells and mRNA expression of MMP-2, caspase-3, and caspase-9 were markedly increased in the scar tissue. The scar elevation index (SEI) was also evidently reduced. Histological findings exhibited significant amelioration of the collagen tissue. These results suggest that OA has the favorable curative effects on formed hypertrophic scars in the rabbit ear model, and the possible mechanism of action is that OA decreases HSFs proliferation and increases HSFs apoptosis by reduction of P311 gene expression and TGF-*β*_1_ production, inhibition of TIMP-1 secretion, enhancement of MMP-2 activity, and subsequently facilitation of degradation of collagen types I and III.

## 1. Introduction

Hypertrophic scarring is a common proliferative disorder of dermal fibroblasts characterized by collagen overproduction and excessive deposition of extracellular matrix (ECM) and occurs in healing wounds elicited by deep burn, inflammatory reactions, and trauma [1]. Itching and pain are often reported by patients with hypertrophic scars, who experience serious functional and cosmetic problems caused by a variety of complications, including compression, sensation of stiffness, loss of joint mobility, and anatomical deformities [2, 3].

There are many treatment choices when exuberant hypertrophic scars produce. Nevertheless, there is no consensus about the best therapeutics to produce complete and permanent improvement of scars with few side effects [4, 5]. It has been generally realized that natural drugs can play a unique role in the prevention and treatment of many diseases. Especially, natural compounds from many plant species have become popular in recent years, whose bioactivities and mechanisms of action are being investigated for human health [6–8].

Oleanolic acid (OA), a naturally occurring triterpenoid compound, has extensive pharmacological activities. OA not only protects the liver effectively from acute liver injury induced by chemical agents, but also defends the liver against fibrosis and cirrhosis caused by chronic liver diseases [9, 10]. Some other pharmacological functions include antiinflammatory, antitumor, hypoglycemic, hypolipidemic, antiatherogenic, and antiobese effects [11–15]. 

We previously screened numerous natural compounds, and OA was found to significantly inhibit the viability of hypertrophic scar fibroblasts and elicit cell apoptosis. The animal experiments indicate that preadministration of OA suppresses hypertrophic scar formation on the rabbit ears [16], suggesting its preventive effects on hypertrophic scarring. However, drugs are more commonly used to treat formed hypertrophic scars clinically and applied less to the prevention of hypertrophic scarring. Furthermore, the good preventive function of a drug does not mean its favorable therapeutic action. 

 As far as we are aware, no investigators have reported the therapeutic effects of OA on produced hypertrophic scars. This study was designed to demonstrate whether OA can alleviate or eliminate formed hypertrophic scars in the rabbit ear model and to explore the possible mechanism of action involved.

## 2. Materials and Methods

### 2.1. Drug Preparation

OA with a purity of 98.58%, obtained as a white powder from Shanxi Yongjian Pharmaceutical Co. Ltd. (Shanxi, China), was mixed with pure vaseline and liquid paraffin ratios of 1 : 7 : 2, 0.5 : 7.5 : 2, and 0.25 : 7.75 : 2 (w : v : v), respectively. The ointment base, consisting of vaseline and liquid paraffin (ratio of 8 : 2), was used as the placebo.

### 2.2. Hypertrophic Scar Rabbit Model

Female New Zealand white rabbits, obtained from Shanghai Si-Lai-Ke Experimental Animal Co., Ltd. (Shanghai, China) and with an initial body weight of 2.5 ± 0.2 kg, were used. A rabbit ear model with hypertrophic scars was established as previously described [17, 18]. All animal treatments were strictly in accordance with the international ethical guidelines and the National Institutes of Health Guide concerning the Care and Use of Laboratory Animals, and the experiments were carried out with the approval of the Animal Experimentation Ethics Committee of the Second Military Medical University.

### 2.3. Grouping and Administration

On postoperative day 29, the scars were randomly divided into five groups, with 16 scars to each group: one control (placebo) group, three OA treatment groups, and one positive group treated with contractubex (abbreviated to Contra., Merz Pharma GmbH, Frankfurt, Germany). The scars in the control group were thinly coated with basic ointment without OA once a day. OA (2.5%, 5%, and 10%, w/v) was applied once daily to the scars in the three treatment groups, and contractubex to the scars in the positive group. Two unwounded rabbits with full-thickness skin on their ears were used as a normal group without treatment. 

### 2.4. Determination of Collagen I and Collagen III

All animals were killed on posttherapeutic day 28 (postoperative day 56), the scar tissue was separated from the rest of the tissue, and the cartilage was removed. The weighed scar tissue was diced and rapidly frozen in liquid nitrogen until used. Once the tissue was removed from the liquid nitrogen, it was maintained at 4°C after thawing. 1 mL of phosphate-buffered saline (pH 7.4) was added to 50 mg of tissue, then the tissue was homogenized and separated by centrifugation at 3000 g and 4°C for 20 min, and the supernatant was collected for assay. 

Collagens I and III were measured by ELISA assay using an appropriate commercial ELISA kit for each, according to the manufacturer's instructions (R&D Systems Inc., Minneapolis, MN, USA) and previous report [18].

### 2.5. RNA Isolation and Fluorescent Quantitative Reverse Transcription-PCR (FQ-RT-PCR)

Total mRNA of the scar tissue was extracted using TriPure Isolation Reagent (Roche Diagnostics, Vilvoorde, Belgium) according to our previous report [19], and the isolated RNA was treated with RNase-free DNase (Promega). Reverse transcription was performed using a cDNA synthesis kit in accordance with the manufacturer's instructions (Applied Biosystems). 

Primer pairs for rabbit genes (MMP1, MMP2, TIMP-1, P311, TGF-*β*_1_, caspase-3, and caspase-9) were designed using the Primer Express design software (Applied Biosystems) and listed in Table 1. The housekeeping gene GAPDH was used as an internal control. FQ-RT-PCR was performed on a real-time PCR instrument (ABI 7900HT, Applied Biosystems) for 40 cycles consisting of denaturation at 95°C for 30 s, annealing at 59°C for 30 s and extension at 72°C for 30 s. All amplifications and detections were carried out in a MicroAmp optical 384-well reaction plate with optical adhesive covers (Applied Biosystems). Relative expression of mRNA (%) = 2^−ΔCT(1,2,3,4,5,6,7)^ × 100%, where CT represents threshold cycle, ΔCT_1_ = CT_(MMP1)_ − CT_(GAPDH)_, ΔCT_2_ = CT_(MMP2)_ − CT_(GAPDH)_, ΔCT_3_ = CT_(TIMP-1)_ − CT_(GAPDH)_, ΔCT_4_ = CT_(P311)_ − CT_(GAPDH)_, ΔCT_5_ = CT_(TGF−*β*1)_ − CT_(GAPDH)_, ΔCT_6_ = CT_(Caspase-3)_ − CT_(GAPDH)_, and ΔCT_7_ = CT_(Caspase-9)_ − CT_(GAPDH)_.

### 2.6. Western Blot Assay

Scar tissue was homogenized in lysis buffer at 4°C for extraction of whole protein. The protein concentration of supernatant was measured. 40 *μ*g of protein from each sample was loaded on 12% polyacrylamide gels, separated by sodium dodecyl sulfate/polyacrylamide gel electrophoresis (SDS/PAGE) and transferred onto PVDF membranes (Millipore, MA, USA) by semidry transfer method. At the same time, *β*-actin protein was added as internal control. Membranes were orderly incubated with 1 : 400 dilution of TGF-*β*_1_ primary antibody for 2 h at room temperature, washed 3 times with PBST for 5 min each time, incubated with 1 : 5000 dilution of TGF-*β*_1_ secondary antibody for 2 h at room temperature, and washed 3 times with PBST for 5 min each time. Blots were detected with an ECL reagent and quantified by measuring the relative intensity compared with the control using image analysis software. 

### 2.7. Detection of Apoptotic Cells by TUNEL Assay

For in situ detection of DNA fragmentation in paraffin-embedded tissue sections, the TUNEL method was performed using the TUNEL Apoptosis Detection Kit (KeyGen Biotech Co., Nanjing, China), following manufacturer's instructions and our previous description [18] The positive cells were counted (×200).

### 2.8. Determination of Scar Elevation Index (SEI)

Scar tissue with cartilage was fixed with 10% buffered formalin for 3 days, embedded in paraffin, sectioned with a dermatome, and stained using hematoxylin-eosin (H&E). Light microscopy was used to examine the degree of scar hyperplasia, which was expressed as SEI. SEI represents the ratio of the scar tissue height to the normal tissue below the hypertrophic scar. A ratio of 1 indicates no difference in the wound area height compared with unwounded skin. 

### 2.9. Masson's Trichrome Staining for Collagen Fibers

Scar tissue was fixed with 10% formalin, embedded in paraffin, sectioned, and stained with Masson's Trichrome Stain Kit, following the manufacturer's instructions (KeyGen Biotech Co., Nanjing, China) and our previous description [18]. 

### 2.10. Statistical Analysis

All results were presented as the mean ± SD. Data were analyzed using SPSS 13.0 statistical package. Data for multiple comparisons were performed by one-way ANOVA followed by Dunnett's test. A value of *P* < 0.05 was considered statistically significant.

## 3. Results

### 3.1. Oleanolic Acid Inhibited the Formation of Collagen I and Collagen III

**Figure 1:**
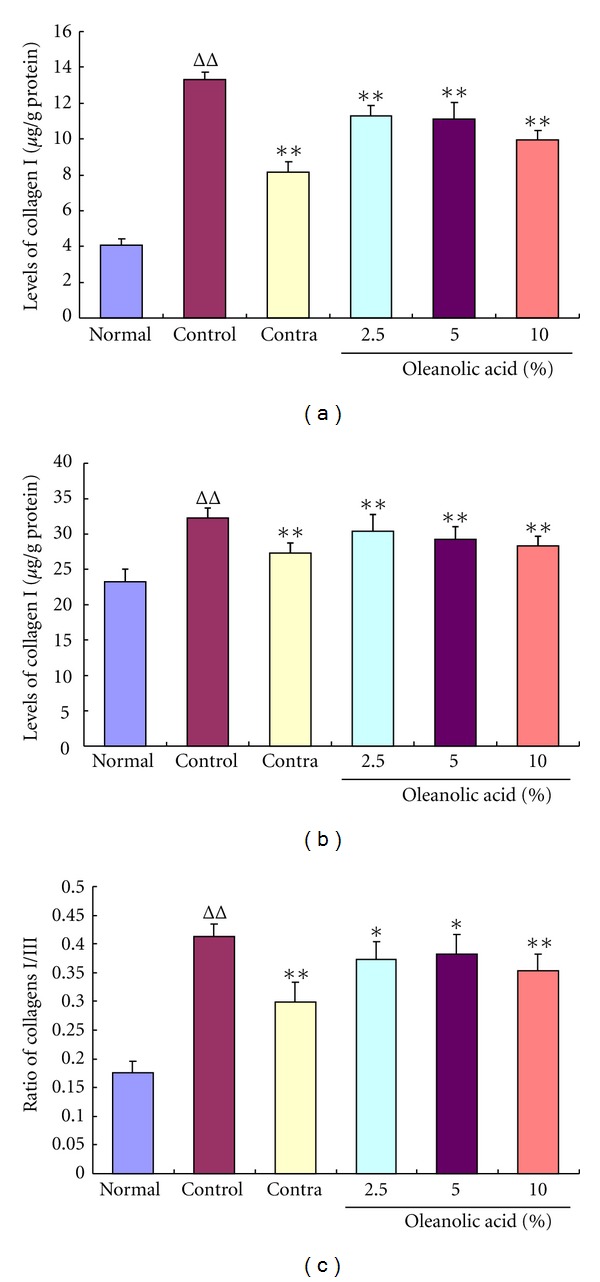
Effects of oleanolic acid (OA) on the levels of collagens I (a) and III (b) and the ratio of collagens I/III (c). The levels of collagens I and III and the ratio of collagens I to III significantly increased in the control group on day 56 postoperation when compared with the normal group. The administration of OA for 28 days reversed these changes dramatically and dose dependently in comparison with the control group. ^ΔΔ^ *P* < 0.01 compared with the normal group; *P < 0.05 and **P < 0.01 compared with the control group. Data are expressed as the mean ± SD. *n* = 16.

The most important ingredients of ECM are collagens, including collagen I and collagen III. As shown in Figures 1(a) and 1(b), the levels of collagens I and III significantly increased in the control group when compared with the normal group. By contrast, in the treatment groups, the levels of collagens I and III decreased evidently after 28 days of the treatment with OA. The ratio of collagens I/III, which reflects tissue flexibility, is evidently higher in the control group than that in the normal group (Figure 1(c)). However, administration of OA for 28 days significantly reduced the ratio compared with the control group.

### 3.2. Oleanolic Acid Regulated the mRNA Expression of MMP-1, MMP-2, TIMP-1, TGF-*β*_1_, P311, Caspase-3, and Caspase-9

MMP-1, TGF-*β*_1_, and P311 play an important role in the formation and development of scars. Their mRNA expression levels significantly ascended in the control group in comparison with the normal group but markedly decreased in the groups of animals treated with the doses of 2.5%, 5%, and 10% OA for 28 consecutive days (Table 2). The mRNA expression level of MMP-2 was not increased in the control group more than in the normal group but enhanced dramatically in the treatment groups, especially in the high dose group. The mRNA expression of TIMP-1, a potent inhibitor of MMPs, was notably enhanced in the control group but dramatically reduced in the treatment group. Activation of caspase-3 and caspase-9 can evidently induce apoptosis of hypertrophic fibroblasts. In this study, their mRNA expression levels decreased in the control group when compared with the normal group while increased dramatically and dose dependently in the treatment groups of animals given the doses of 2.5%, 5%, and 10% OA, respectively, for 28 days.

### 3.3. Oleanolic Acid Reduced the Protein Expression of TGF-*β*_1_

**Figure 2:**
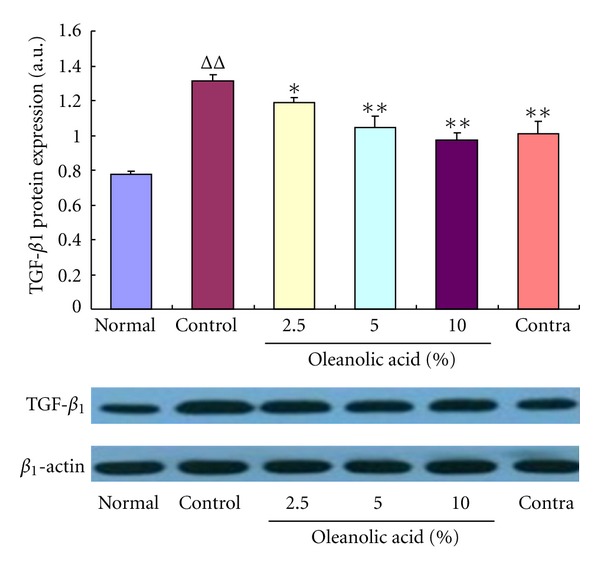
Effects of OA on TGF-*β*_1_ protein expression in the scar tissue. The protein expression level of TGF-*β*_1_ was significantly increased in the control group of scar tissue but evidently decreased following administration of OA (2.5%, 5%, and 10%) for 28 consecutive days. ^ΔΔ^ *P* < 0.01 compared with the normal group; *P < 0.05 and **P < 0.01 compared with the control group. Data are expressed as the mean ± SD. *n* = 3.

TGF-*β*_1_ can promote tissue repair and regeneration, but its excessive expression facilitates scar formation. After 56 postoperative days, the protein expression level of TGF-*β*_1_ was evidently enhanced in the control group in comparison with the normal group (Figure 2). However, this protein expression was significantly reduced by OA treatment for 28 successive days, which is consistent with the mRNA expression. 

### 3.4. Scar Elevation Index

**Figure 3:**
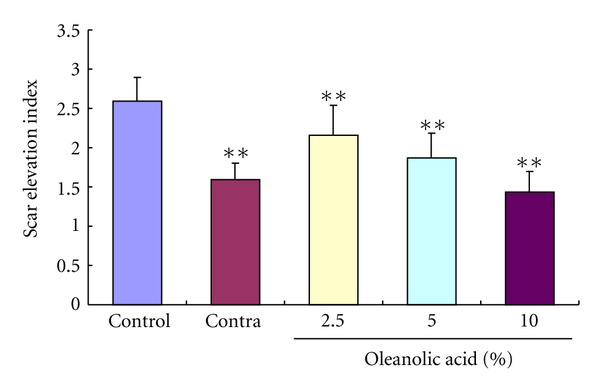
Effects of OA on the scar elevation index (SEI). The degree of scar hypertrophy can be reflected by SEI, which represents the ratio of total scar connective tissue area to the area of underlying dermis. After treatment with OA for 28 days, the formed hypertrophic scars decreased in a dose-dependent manner. **P < 0.01 versus the control group. Data are expressed as the mean ± SD. *n* = 16.

On day 56 postwounding, there was significant hypertrophic scarring in the control group with the mean scar elevation index (SEI) of 2.59 ± 0.47 (Figure 3). Consecutive administration of contractubex or OA for 28 days since 29 postwounding markedly and dose dependently inhibited scar hyperplasia in the treatment groups with the mean SEI of 1.59 ± 0.21, 2.16 ± 0.38, 1.87 ± 0.32, and 1.44 ± 0.26, respectively. 

### 3.5. Quantification of TUNEL Positive Cells

**Figure 4:**
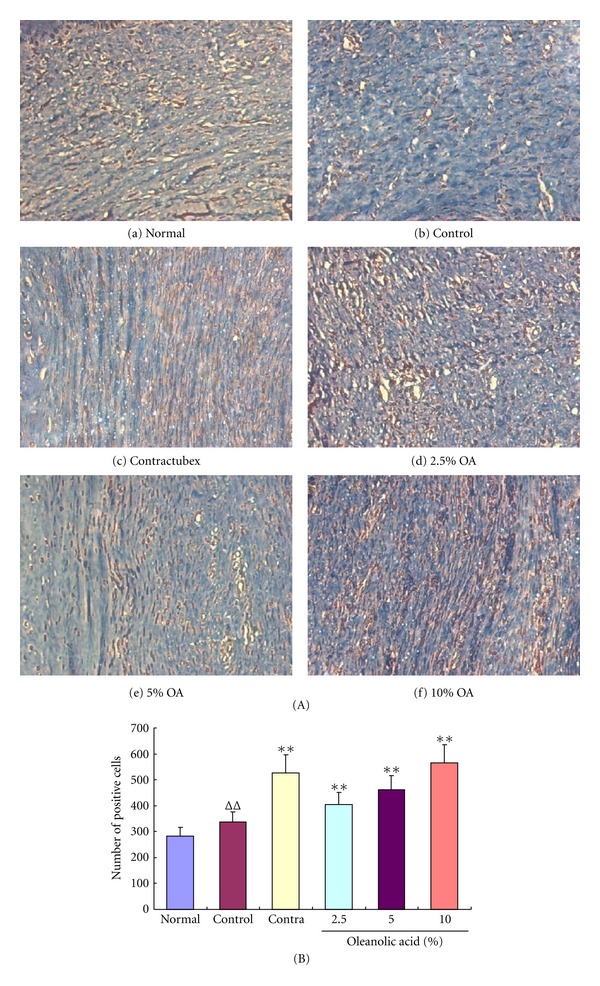
OA increased the number of TUNEL positive cells. (A) Fragmented DNA was displayed as yellow grains, which indicate apoptotic cells. After 56 operative days, the amount of TUNEL positive cells markedly increased in the control group of scar tissue (b) in comparison with the normal group (a). Nevertheless the treatment with OA for 28 days further increased the number of TUNEL positive cells ((d)–(f)) when compared with the control group. (B) The number of TUNEL positive cells was revealed in the different groups. ^ΔΔ^ *P* < 0.01 compared with the normal group; **P < 0.01 compared with the control group. Data are expressed as the mean ± SD. *n* = 16.

The sections from rabbit ears subjected to OA were stained for visualization of fragmented DNA, which was displayed as yellow grains and indicated apoptosis cells (Figure 4(A)). One of the characteristics of hypertrophic scars is abnormal proliferation of dermal fibroblasts, but OA significantly induced apoptosis of scar fibroblasts in this study. As shown in Figure 4(B), the number of TUNEL positive cells in the control group was higher than that in the normal group, indicating an increase in apoptosis of scar fibroblasts, but significantly lower than that in the groups treated with the doses of 2.5%, 5% and 10% OA for 28 consecutive days, suggesting that OA elicits more apoptosis of scar fibroblasts.

### 3.6. Masson's Trichrome Staining Findings

**Figure 5:**
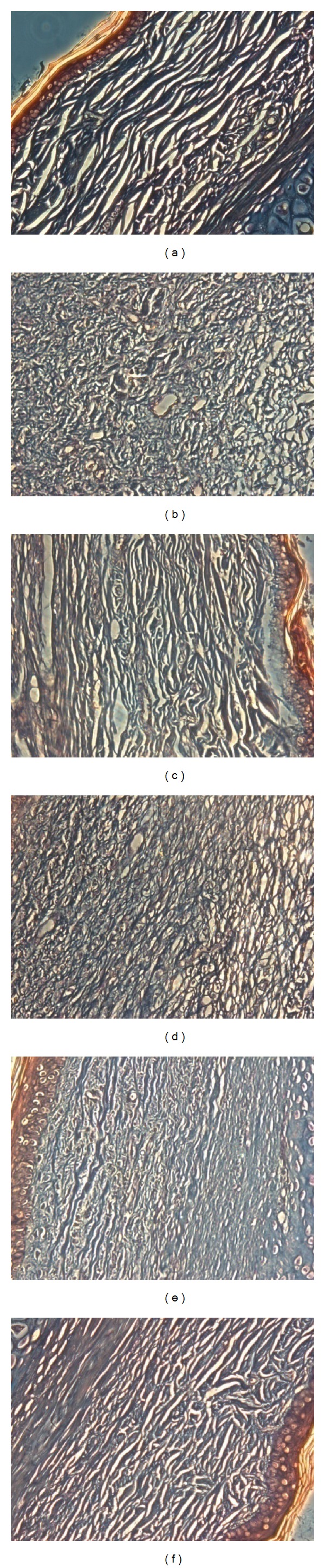
Masson's trichrome staining findings for collagen. (a) Unwounded (normal) skin with regularly arranged small collagen fibers. (b) In the control group treated with ointment base, collage fibers are thicker, denser, and disorganized, the amount of which is far more than that in the normal group. (c) Contractubex. ((d)–(f)) After treatment with (d) 2.5%, (e) 5%, and (f) 10% oleanolic acid (OA) for 28 days, collagen fibers were arranged more regularly and sparse than those of the scar tissue in the control group. Original magnification ×200.

Overproduction of collage is another characteristic of hypertrophic scars. Masson staining of scar tissue was carried out on day 56 postwounding. Light microscopic examination revealed typical features of collagen fibers in scar tissue (Figure 5(b)) in the control group compared with unwounded dermal tissue (Figure 5(a)). The collagen bundles were thicker, denser, disorganized, and more abundant. By contrast, collage fibers were decreased, thinner, and more regularly ranged in the groups treated with OA, which was found to be dose dependent (Figures 5(d), 5(e), and 5(f)).

## 4. Discussion

Hypertrophic scars possess the properties of overproduction of fibroblasts and excessive deposition of collagen. Hypertrophic scar fibroblasts (HSFs) have a higher capacity for proliferation, cytokine production, and collagen synthesis than that of normal fibroblasts [20]. Thus, inhibition of proliferation and induction of apoptosis of HSFs are the major therapeutic modalities for hypertrophic scars. Our previous investigations have displayed that OA evidently causes HSFs apoptosis from human skin tissue. In this study, in situ detection of cell apoptosis was applied to confirmation of OA-facilitated apoptosis of HSFs in scar tissue. It is interesting that OA treatment for 28 days markedly and dose dependently increased the number of TUNEL positive cells and the mRNA expression of caspase-3 and -9 in the scar tissue of rabbit ears, suggesting that OA is responsible for more apoptosis of scar fibroblasts.

Collagen, secreted mainly by fibroblasts and the most important constituent of extracellular matrix (ECM), is the principal structural protein holding the skin. Although collagen I is most abundant in normal human skin, our findings showed that the content of collagen III was higher than that of collagen I in normal rabbit skin, indicating different composition proportion of collagen I to collagen III between human skin and rabbit skin. After 56 days of operation, the levels of collagens I and III evidently increased in the hypertrophic scar tissue of rabbit ears in the control group compared with the normal group, but OA reversed these changes dramatically and dose dependently. Furthermore, the ratio of collagens I/III was also notably decreased by OA treatment for 28 days. Pathological examination showed that collagen fibers evidently decreased in OA treatment groups compared with the control group. At the same time, administration of OA for 28 days markedly decreased the scar elevation index (SEI) of rabbit ears. These results indicate that OA not only decreases scar hyperplasia, but also increases scar compliance. However, the mechanism of action is unclear.

We further observed the mRNA expression levels of matrix metalloproteinase-1 (MMP-1), matrix metalloproteinase-2 (MMP-2), metallopeptidase inhibitor-1 (TIMP-1), transforming growth factor beta 1 (TGF-*β*_1_), and P311 in the scar tissue, which are closely related to overproduction of fibroblasts and excessive deposition of collagen. Wound repair involves cell migration, proliferation, and tissue remodeling. These ordered and regulated processes are facilitated by matrix-degrading proteases. Collagenases are the only known enzymes able to initiate breakdown of the interstitial collagens, such as types I, II, III, and IV. MMP-1 plays a key role in the remodelling that occurs constantly in both unwounded and diseased conditions [21] and is the key enzyme in the degradation of type I and type III collagens in scars. MMP-2 can also degrade collagen type I, although degradation of collagen type IV is its main function [22]. TIMP-1 can bind to almost all MMP active sites, thereby irreversibly inhibiting the enzyme activity [23, 24]. 

On the other hand, considerable evidence indicates that many cytokines are important components in the process of wound repair and scar formation. TGF-*β*_1_, one of the most intensively investigated molecules associated with many types of fibrosis, stimulates infiltration of inflammatory cells and facilitates fibroblast proliferation, angiogenesis, and synthesis of ECM, while a persistent autocrine loop of TGF-*β*_1_ redounds to hypertrophic scarring [25].

It was observed in the current study that the mRNA expression of MMP-1, TIMP-1, and TGF-*β*_1_ notably increased in the control group of scar tissue. Although these findings are consistent with previous results obtained from human skin hypertrophic scars [26, 27], it seems to be difficult to understand. Presumably, there exists a feedback regulating mechanism. Excessive TGF-*β*_1_ accelerated overproduction of scar fibroblasts, while scar fibroblasts further secreted more MMP-1 to degrade collagen protein. At the same time, TIMP-1 overproduced by scar fibroblasts inhibited MMP-1 activity through combination with its active sites. It is interesting that OA treatment for 28 days markedly decreased their mRNA expression levels. Unlike the previous parameters, enhanced MMP-2 expression does not appear in the control group of scar tissue, similar to the previous report [28]. But OA, especially at high dose, dramatically increased MMP-2 expression level, suggesting that the regulation of MMP-2 probably plays an extremely important role in the process of OA inhibition of hypertrophic scarring.

The expression of P311 mRNA and protein commonly does not exist in normal human dermal tissue but is markedly upregulated in human hypertrophic scar tissue [29, 30]. Interference of P311 gene expression can decrease TGF-*β*_1_ mRNA expression in human hypertrophic scar fibroblasts [30]. Nevertheless, P311 mRNA expression was clearly detected in the normal tissue of rabbit skin in this study, which is likely associated with different species of animals. Similarly, P311 mRNA expression was significantly increased in the control group of scar tissue but obviously reduced in OA treatment groups, revealing that OA inhibits TGF-*β*_1_ mRNA expression probably via downregulation of P311 mRNA expression. 

Scar fibroblasts synthesize collagens I and III, which are the major components of ECM, and also excrete MMP-1, MMP-2, TIMP-1, TGF-*β*_1_, and P311, which regulate the synthesis and degradation of collagens. On the basis of these results, we hypothesize that OA alleviates formed hypertrophic scars mainly through inhibition of HSFs proliferation and induction of HSFs apoptosis, as the abnormal biological behavior of fibroblasts plays a central role in the process of hypertrophic scar formation and development [31]. 

## 5. Conclusion

In summary, OA mitigates produced hypertrophic scars in the rabbit ear model, probably via inhibition of HSFs proliferation and induction of HSFs apoptosis, which further reduces TGF-*β*_1_ production by inhibition of P311 gene expression, lessens TIMP-1 secretion, increases MMP-2 activity, and subsequently upregulates degradation of collagens I and III. OA may be a potential drug in the treatment of human hypertrophic scars. 

## Figures and Tables

**Table 1:** Primers used in real-time RT-PCR analysis.

Gene	Primer sequence	Species	Amplicon size (bp)
MMP1	Forward: 5′ GAAGCCAGATGCTGAAAC 3′	Rabbit	237
	Reverse: 5′ TGACCCTTGGAGACTTTG 3′		
MMP2	Forward: 5′ CTGAAGAGCGTGAAGGTTGG 3′	Rabbit	221
	Reverse: 5′ TGAGGGTTGACGGGATTG 3′		
TIMP-1	Forward: 5′ TCCCACAAATCCCAGAAC 3′	Rabbit	229
	Reverse: 5′ CTGTCCACAAGCAATGAG 3′		
TGF-*β*_1_	Forward: 5′ CACCCAGTGACACAGATGAG 3′	Rabbit	248
	Reverse: 5′ ATGTTGAGCCCGTTCCAG 3′		
P311	Forward: 5′ CGTCCCAAAGGAAGTGAAC 3′	Rabbit	115
	Reverse: 5′ TCAGAAAGAGTGGCGGTAG 3′		
Caspase-3	Forward: 5′ TAAGCCACGGTGATGAAG 3′	Rabbit	136
	Reverse: 5′ CGGCAAGCCTGAATAATG 3′		
Caspase-9	Forward: 5′ GAACTTCAGCAGCACCTC 3′	Rabbit	130
	Reverse: 5′ CATTTCCTTGGCGGTCAG 3′		
GAPDH	Forward: 5′ CTCCTGCGACTTCAACAGTG 3′	Rabbit	172
	Reverse: 5′ TGAGGGCTCTTACTCCTTGG 3′		

**Table 2:** Effects of oleanolic acid (OA) on the mRNA expression levels of MMP-1, MMP-2, TIMP-1, TGF-*β*_1_, P311, caspase-3, and caspase-9.

Group	Scar (*N*)	Dose (%)	MMP-1 (%)	MMP-2 (%)	TIMP-1 (%)	Caspase-3 (%)	Caspase-9 (%)	P311 (%)	TGF-*β*_1_ (%)
Normal	16	—	0.055 ± 0.010	0.209 ± 0.031	0.011 ± 0.001	0.721 ± 0.084	0.654 ± 0.075	0.121 ± 0.033	0.383 ± 0.083
Control	16	Basic ointment	1.125 ± 0.147^ΔΔ^	0.222 ± 0.033	0.054 ± 0.008^ΔΔ^	0.441 ± 0.078^ΔΔ^	0.431 ± 0.079^ΔΔ^	0.436 ± 0.090^ΔΔ^	3.248 ± 0.482^ΔΔ^
Contra	16	10	0.314 ± 0.047**	1.589 ± 0.239**	0.014 ± 0.005**	1.101 ± 0.165**	1.266 ± 0.281**	0.172 ± 0.056**	0.637 ± 0.134**
OA	16	2.5	0.676 ± 0.216**	1.689 ± 0.267**	0.024 ± 0.009**	0.621 ± 0.155*	0.719 ± 0.195**	0.298 ± 0.067**	1.703 ± 0.242**
OA	16	5	0.302 ± 0.134**	2.486 ± 0.235**	0.030 ± 0.002**	0.907 ± 0.157**	0.934 ± 0.186**	0.253 ± 0.049**	1.481 ± 0.272**
OA	16	10	0.250 ± 0.063**	7.014 ± 0.790**	0.023 ± 0.006**	1.087 ± 0.134**	1.368 ± 0.256**	0.147 ± 0.041**	0.655 ± 0.157**

^ΔΔ^ *P* < 0.01 compared with the normal group; *P < 0.05 and **P < 0.01 compared with the control group; Data are expressed as the mean ± SD.
